# Identification and Translocation of Potentially Toxic Elements in Sorghum Plants Grown in Central Mexico

**DOI:** 10.3390/toxics14040290

**Published:** 2026-03-28

**Authors:** Luis Eduardo Herrera-Figueroa, Francisco Rodríguez-González, Rodolfo Figueroa-Brito, Santos Margarito Herrera-Cadena, Silvia Viridiana Vargas-Solano, Alex Osorio-Ruiz, Miguel Mauricio Correa-Ramírez, Carlos Enrique Ail-Catzim, Pedro Joaquín Gutiérrez-Yurrita, Juan Alberto Alcántara-Cárdenas

**Affiliations:** 1 Centro de Desarrollo de Productos Bióticos, Instituto Politécnico Nacional, Carretera Yautepec-Jojutla Km. 6, Calle CEPROBI No. 8, San Isidro, Yautepec 62739, Morelos, Mexico; lherreraf1700@alumno.ipn.mx (L.E.H.-F.); rfigueroa@ipn.mx (R.F.-B.); sherrerac@ipn.mx (S.M.H.-C.); svvargass@ipn.mx (S.V.V.-S.); 2 Estancia Posdoctoral SECIHTI, Centro de Desarrollo de Productos Bióticos, Instituto Politécnico Nacional, Carretera Yautepec-Jojutla Km. 6, Calle CEPROBI No. 8, San Isidro, Yautepec 62739, Morelos, Mexico; aosorior1300@alumno.ipn.mx; 3 Centro Interdisciplinario de Investigación para el Desarrollo Integral Regional Durango, Instituto Politécnico Nacional, Sigma 119, 20 de Noviembre II, Durango 34220, Durango, Mexico; mmcorrear@ipn.mx; 4 Instituto de Ciencias Agrarias, Universidad Autónoma de Baja California, Carretera a Delta s/n, Mexicali 21705, Baja California, Mexico; carlos.ail@uabc.edu.mx; 5 Centro Interdisciplinario de Investigaciones y Estudios Sobre Medio Ambiente y Desarrollo, Instituto Politécnico Nacional, Calle 30 de Junio de 1520 s/n, Barrio la Laguna Ticomán, Alcaldía Gustavo A. Madero, Mexico City 07340, Mexico; pgutierrezy@ipn.mx (P.J.G.-Y.); jaalcantarac@ipn.mx (J.A.A.-C.)

**Keywords:** sorghum crops, translocation in tissues, anthropogenic pollution, volcanic pollution, agrochemical contamination, PTEs

## Abstract

Contamination of agricultural soil by potentially toxic elements (PTEs) can be caused by volcanic emissions and the use of agrochemicals; this threatens human food security, as PTEs can be transferred from the soil to plant tissues. Sorghum is the fifth most important cereal crop worldwide, and Mexico is one of the countries with the highest sorghum production. However, these crops are vulnerable to pests; thus, agrochemicals are applied to eliminate them. In this study, the identification and translocation of PTEs into sorghum plants grown in urban and volcanic areas of central Mexico were evaluated. Sorghum plants and soil samples were collected at four sites (S1, S2, S3, and S4) in these areas. The concentrations of PTEs in the soil samples and in the different tissues of the sorghum plants were determined by inductively coupled plasma optical emission spectroscopy. It was found that these sites are contaminated with PTEs, which were attributed to volcanic emissions and anthropogenic activities. In addition, the translocation factor values for zinc, nickel, and manganese showed that these PTEs were retained in the roots of the sorghum plants; however, the average concentrations of these PTEs in the grains of the plants were higher than the translocation factor values. This result indicates that the aerial parts of the sorghum plants could have been contaminated with PTEs from the air, which could then enter humans throughout the food chain.

## 1. Introduction

Contamination of agricultural soils plays a key role in sustainability and food security [1]. This contamination is caused by natural and anthropogenic sources, with the former including forest fires, volcanic eruptions, and acid rain, and the latter including sewage and industrial waste discharges, as well as poor agricultural practices [2,3,4,5,6,7]. In addition, these sources of contamination can affect trace element concentrations and incorporate potentially toxic elements (PTEs) into soils [8,9]. PTEs are chemical elements composed of metals, metalloids, and non-metals, and can therefore be grouped as pollutants with a high level of toxicity to living organisms, as they can persist in the environment because they are not degradable. Some elements are considered essential for the growth and metabolism of living beings, such as calcium (Ca), copper (Cu), iron (Fe), magnesium (Mg), manganese (Mn), and zinc (Zn); however, in high concentrations, they could have harmful effects on human health, which is why some authors classify them as PTEs. On the other hand, PTEs such as arsenic (As), cadmium (Cd), lead (Pb), chromium (Cr), cobalt (Co), nickel (Ni), and titanium (Ti), among others, are considered non-essential due to their high toxicity, even at low concentrations in the environment [10,11,12]. These PTEs, when present in soil and water, can accumulate in plant and animal tissues, contaminating food and, when consumed, can cause irreversible damage to human health [13].

The agricultural sector is essential for human survival and is of great importance to the economy of countries. The success of the agricultural sector depends entirely on soil quality, physiological conditions, and total production [3,5]. However, continuous changes in agricultural soils due to the use of synthetic agrochemicals and the discharged wastewater from mines and factories can alter the system, leading to increased soil contamination and decreased soil quality [3,13,14]. Moreover, the combustion of fuels by cars also contributes to the accumulation of PTEs in agricultural soils near roads and highways [3,8]. In general, this environmental stress creates a critical need to safeguard the quality of the soil in agricultural fields. In addition, non-essential PTEs represent an environmental threat due to their ability to cause poisoning in food and agricultural crops [3,5,8]. Daily food consumption is one of the main routes of entry of PTEs into human tissues as they tend to be transferred from the soil to plants where they accumulate before entering humans through food supply chains, causing immediate or indirect damage to human health [3,5,8,13].

One commonly consumed crop is sorghum, which is the fifth most important cereal crop worldwide. It is of African origin according to reports from Ethiopia around the year 5000 B.C, from India in 4000 B.C, and from China and southern Africa in approximately 1500 B.C [15,16]. This crop is characterized by its C4 metabolism, which allows it to withstand hot, arid, or semi-arid climatic conditions. Mexico is the fourth largest sorghum producer in the world, accounting for 7% of the total production. It is the third most important crop in Mexico, with 4200 MT produced annually [17,18]. Sorghum is considered a dual-purpose crop, since the grain is highly valued and the stubble is used in livestock feed and, in some cases, in the poultry industry [19]. In West Africa, the fodder is used for roofing [15]. The sorghum industry in Mexico is divided into two sectors: modern large-scale agriculture (used industrially in livestock feed and bioethanol production) and small-scale, traditional agriculture (self-consumption) [20], with the latter being the predominant sector in Mexico. Nevertheless, its production has faced challenges worldwide due to biotic stress factors such as the presence of pests and diseases, as well as land-use changes [15].

The state of Morelos, located in central Mexico and considered one of the main sorghum-producing states in the country, has been affected in terms of yield and crop quality due to the sugarcane aphid plague “*Melanaphis sacchari* Zetner”, one of the most common pests in sorghum crops worldwide, since 2015 [21]. In Mexico, the sugarcane aphid has caused sorghum crop losses of more than 60%, leading producers to resort to conventional management, such as synthetic agrochemicals (phosphate fertilizers and pesticides based on neonicotinoids, dimethoate, cypermethrin, and glyphosate, among others), to mitigate the impact of this pest and stabilize production [5,22,23]. Furthermore, it has been reported that PTEs in crop fields could come from volcanic ash, fossil fuel combustion, the mining industry, as well as the excessive use of agrochemicals, among other sources [12,24,25,26,27,28]. Therefore, the objective of this study was to identify and quantify PTEs in sorghum plants grown in urban-volcanic areas of central Mexico, and their translocation into different tissues (roots, stems, and grains).

## 2. Materials and Methods

### 2.1. Study Area

The state of Morelos is located in central Mexico (latitude: 19°07′54″ N, 18°19′57″ S; longitude: 98°37′59″ E, 99°29′40″ W); it is bordered by the state of Mexico and Mexico City to the north, the Sierra Neovolcanica (Iztaccihuatl and Popocatepetl volcanoes, the latter of which is an active volcano) to the northeast, Puebla state to the east, Guerrero and Puebla states to the south, and Guerrero state and the state of Mexico to the west [29]. It has an area of 4879 km^2^ and represents 0.2% of the Mexican territory [30]. It has a warm sub-humid climate with an average annual temperature of 21.5 °C and an annual rainfall of 900 mm. It contains 11 soil types, with leptosol being the predominant type [30,31]. The state of Morelos possesses great natural and mineral wealth, which has allowed for the establishment and development of various industries in recent years [32]. According to the SGM [33], the state has more than 100 mines and a thermoelectric plant, as well as high agricultural activity. The highest sorghum production in the state of Morelos was achieved in the municipalities of Yecapixtla and Tepalcingo at 4350 ha and 3216 ha, respectively, while Tepoztlan had an average production of 26 ha and Xoxocotla had the lowest production, according to SIAP [17]. These municipalities were selected to carry out the present study.

### 2.2. Soil and Sorghum Plant Sample Collection

**Figure 1:**
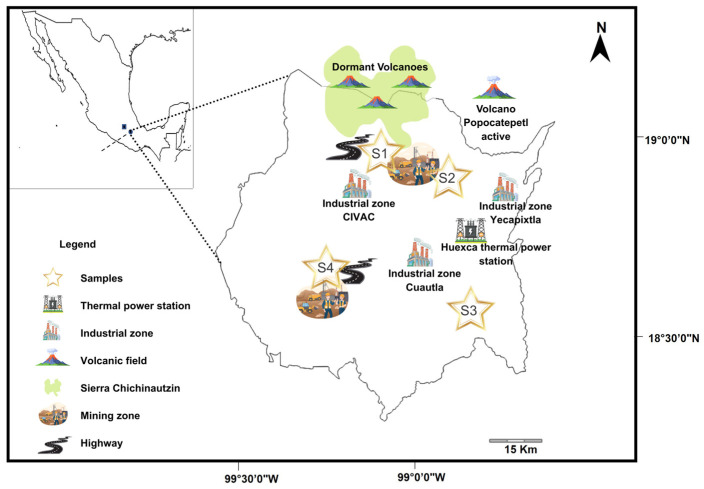
Map of the state of Morelos showing the main sources of PTEs contamination.

The municipalities selected for the study were Tepoztlan (S1), Yecapixtla (S2), Tepalcingo (S3), and Xoxocotla (S4). In each municipality, a sorghum crop with an area of 0.5 hectares, and under conventional management was chosen (Figure 1). At each site, soil and sorghum plant samples were collected at maturity (3 months after agricultural inputs were applied to the crops) between the summer and fall of 2022. The collection of the samples was carried out using a five-hole scheme [34], which consisted of selecting five surfaces or areas of 1 × 1 m^2^ distributed throughout the crop, and from each area, five samples of 0.2 kg (wet weight) of soil were collected from a depth of 15 to 20 cm. Subsequently, these were mixed to obtain 1 kg of a homogeneous soil sample per area and a total of 5 kg of soil was collected for each site [35] (see Supplementary Materials). In addition, three sorghum plants were randomly collected from each area (1 × 1 m^2^) of each site, thus obtaining 15 plants per site. The plants were stored in polyethylene bags and transported to the laboratory, where they were stored in a cold chamber (4 °C) until analysis.

### 2.3. Sample Analysis

Sorghum plants from each of the studied municipalities were washed with running water and then with deionized water to remove surface impurities. The sorghum plants and the soil samples were placed in plastic containers and then covered with a cloth (pore size of 0.042 mm); subsequently, all samples were dried in the open air (average temperature: 34 °C) for 10 days. After this time, the plant samples were divided into root, stem, and grain samples for further analysis. Next, each part of the plant was ground using a conventional electric grinder (model 1000A, LEJIEYIN, Wuhan, China) and sieved through a 10-mesh (2 mm) screen. The soil samples were also sieved using the same mesh to homogenize the particle size of all samples.

#### Physicochemical Analysis of Soil Samples

The determination of the collected soil samples’ pH, organic matter (OM) content, and cation exchange capacity (CEC) was carried out using methods AS-02, AS-07, and AS-12 of NOM-021-RECNAT-2000 [36], respectively. The concentration of PTEs (As, Cd, Co, Cr, Cu, Fe, Mn, Ni, Pb, Ti, and Zn) in the soil samples and the different parts of sorghum plants was measured according to EPA Method 6010D [37]. To a Berzelius beaker (Pyrex, Corning, Steuben County, NY, USA), 1 g of sample was added along with 2 mL of ultrapure nitric acid (HNO_3_, 67–70% *w*/*w*, J.T. Baker, Avantor, Radnor, PA, USA), 0.5 mL of ultrapure hydrochloric acid (HCl, 33–36% *w*/*w*, J.T. Baker, Avantor, Radnor, PA, USA), and 5 mL of ultrapure hydrogen peroxide (H_2_O_2_, 30% *w*/*w*, J.T. Baker, Avantor, Radnor, PA, USA) and pre-digestion was performed for 24 h; then, 5 mL of hydrogen peroxide was added and plate digestion (Cimarec, Thermo Scientific, Waltman, MA, USA) was performed at 90 °C for 4 h. Subsequently, the samples were cooled to room temperature, filtered (Grade 44, Whatman, Maidstone, Kent, UK), and brought to a final volume of 50 mL with Milli Q ultrapure water (Merck Millipore, Burlington, MA, USA) in a volumetric flask (Pyrex, Corning, New York, NY, USA). The concentration of PTEs in the samples was determined using an ICP-OES (inductively coupled plasma optical emission spectroscopy) instrument (ICP-OES 8300 DV, Perkin Elmer, Waltman, MA, USA). The operating conditions for this method are presented in Table S1 (Supplementary Materials). The precision of the analysis was maintained by using a certified high-purity standard with 21 elements at a concentration of 100 µg mL^−1^ in 4% HNO_3_ + Tr HF (SRM 3100, NIST, Gaithersburg, MD, USA); the reliability of the analysis was ensured by testing blank samples (water and the ultrapure reagents used in the determination) every 10 readings. The recovery rate for the studied elements was between 89 and 101%; each determination was performed in triplicate. The limit of detection and limit of quantification for each element are presented in Table S2 (Supplementary Materials).

### 2.4. Determination of Contamination Factors

The contamination factor (*CF*), enrichment factor (*EF*), bioaccumulation factor (*BAF*), and translocation factor (*TF*) were used to quantitatively describe the variation in PTEs contamination in the soils and different parts of the collected sorghum plants. The references used in the study were the average background values (*BV*) of PTEs concentrations in uncontaminated soils from the United States of America, Spain, and other parts of the world [38,39].

#### 2.4.1. Contamination Factor

The contamination factor (*CF*) is commonly employed to assess soil PTE contamination [40]. The *CF* is calculated using the following equation:

(1) $$CF=\frac{M_{S}}{M_{BV}}$$

where *M_S_* is the measured PTEs concentration in the sample, and *M_BV_* is the background PTEs concentration. Based on the contamination factor, soils are classified as Class I (*CF* ˂ 1, pristine), Class II (1 ≤ *CF* ˂ 3; moderately contaminated), Class III (3 ≤ *CF* ˂ 6; considerably contaminated), and Class IV (*CF* > 6, highly contaminated) [39,40].

#### 2.4.2. Enrichment Factor

The enrichment factor *(EF*) is commonly used to determine soil’s degree of contamination with PTEs of interest and can be used to infer the origin of the PTEs [41]. In addition, it considers naturally occurring and abundant elements, such as iron, which is used for normalization as its distribution has not been associated with that of other PTEs [42]. Therefore, in this study, Fe was used for the normalization of the PTEs levels in the soil samples and the different plant parts to calculate the *EF*:

(2) $$EF=\frac{\left(M_{s})({Fe}_{BV}\right)}{\left(M_{BV})({Fe}_{s}\right)}$$

where *Fe_BV_* and *Fe_S_* represent the background value and the concentration of Fe in the soil sample, respectively. If *EF* < 1, it indicates that the analyzed PTEs may be of natural origin, whereas values between 1 < *EF* ≤ 1.5 indicate that they may be of anthropogenic origin, and values of 1.5 < *EF* ≤ 3, 3 < *EF* ≤ 5, 5 < *EF* ≤ 10, and *EF* > 10 are considered evidence of minor, moderate, severe, and very severe anthropogenic modification, respectively [39,40].

#### 2.4.3. Bioaccumulation Factor

The bioaccumulation factor (*BAF*) is defined as the capacity of a plant to absorb and store PTEs from the soil in its different tissues [1,43]. It is calculated as follows:

(3) $$BAF=\frac{M_{plant}}{M_{soil}}$$

where *M_plant_* represents the concentration of a specific metal ion in the whole plant or in a specific plant tissue, and *M_soil_* is the concentration of PTEs present in the soil. *BAF* values < 0.1 indicate that the plant cannot absorb PTEs. *BAF* values ≤ 1 indicate that the plant is suitable for phytostabilization, and has the capacity to absorb PTEs, but they are not stored in the plant’s tissues. *BAF* values > 1 indicate that there is uptake and storage of PTEs in plant tissues, which can be employed for phytoremediation [44].

#### 2.4.4. Translocation Factor

The translocation factor (*TF*) reflects the efficiency of the plant in transporting PTEs from underground tissues (root) to aerial tissues [1,13]. It is calculated using the following equation:

(4) $$TF=\frac{M_{height}}{M_{root}}$$

where *M_height_* is the concentration of a specific PTE in one aerial part (stem or grains) of the plant, and *M_root_* is the concentration of the PTE in the underground part (roots) of the plant. *TF* values < 0.5 indicate that the plant accumulates PTEs in its roots, while *TF* values > 1 indicate that the plant can transport PTEs from its underground tissues to its aerial tissues [45].

### 2.5. Statistical Analysis

Statistical analysis of soil physicochemical characteristics and total PTEs concentrations in the crop fields was performed to calculate mean values and standard deviations. Statistically significant differences were examined using one-way ANOVA and Statistica software, version 12.0 (2013) (StatSoft Inc., Tulsa, OK, USA). When a statistically significant difference was found, a Tukey post hoc test (*p* < 0.05) was performed for pairwise comparisons of the mean values of the data.

## 3. Results and Discussion

### 3.1. Physicochemical Parameters of Sorghum Growing Soils

The physicochemical parameters (pH, organic matter content, and cation exchange capacity) of the soil samples collected from the different sorghum fields are presented in Table 1. The samples presented pH values between 4.9 and 6.6, indicating that they are strongly acidic (<5.0), moderately acidic (5.1 to 6.5), and neutral (6.6 to 7.3) [36]. These pH values may be related to the application of agricultural inputs to the crop fields for soil nutrition [3]; for example, ammonia nutrition induces soil acidification, which is mediated by plant roots [46]. Crop soils with acidic pH values can cause cultivated plants to increase uptake of PTEs compared to alkaline soils (pH ~7.9) [46,47] since PTEs have lower solubility and mobility in alkaline soils [40]. Thus, our result indicates that the studied sorghum plants came from sites with strongly acidic soils, allowing them to absorb PTEs.

The percentage of organic matter (OM) in the collected soil samples ranged between 1.75 and 4.09%; this result may be related to the lack of organic inputs (compost, organic manures, and earthworm humus) to the sorghum crops [13] as these percentages are considered low (OM ˂ 6.0%) [13]. In a similar context, do Nacimiento et al. [47] found that maize plants collected from different cultivation sites with acidic soils and low organic matter percentages (less than 6.0%) contained a higher amount of PTEs than the amounts in cultivated soils.

The soil samples showed cation exchange capacities of 10.4–17.2 Cmol/kg, indicating that negative charges are present on the surface of the minerals and organic components of the soils, and that the soils can retain cations, which are nutrients required by plants [48]. The CEC values were between 10 and 14 Cmol/kg, which are considered low and indicate a weak ability to retain nutrients and a low percentage of organic matter according to the classification of NOM-021-RENACT-2002 [36,48]. This is consistent with the OM percentages determined for the sorghum crop soils.

### 3.2. PTEs Identified in Sorghum Crop Soils

#### 3.2.1. Identification and Concentration of PTEs

The concentration of each PTE identified, and the total PTE concentration in the different soils of the sorghum crops, as well as the safe values and permissible limits for each of PTEs, are presented in Table 2. The concentrations of As, Cd, Co, Cr, Mn, Ni, Ti, and Zn were lower than the values reported in the literature for safe soils for humans and the environment [38,49]. Some PTEs, namely Cd, Co, Cu, Fe, Mn, Ni, and Zn, are available in soil and are essential as micronutrients, but excess uptake by plants may result in toxic effects and could reach humans and animals through the food chain [13,50,51].

The concentration of these PTEs may have been influenced by the irregular application of inorganic fertilizers to sorghum crops (more than twice per year). Tóth et al. [52] suggested that the application of synthetic agrochemicals to agricultural fields promotes the presence of Cd, Cr, Ni, Pb, and Zn in varying concentrations in crop soils. Furthermore, the frequent use of these agrochemicals in agricultural systems can increase the levels of trace metals, namely Cd and Pb, in crop soils [53]. On the other hand, the application of animal manure has been reported to enrich crop soil by incorporating Co, Cu, Mn, and Zn. Thus, the PTEs contamination level of agricultural soils will depend on the frequency of application of agrochemicals and the physicochemical characteristics of the soil [54].

It is not known whether Ti influences plant growth, but at high concentrations, it has been reported to be phytotoxic to plants [55]. Some authors have reported that the incorporation of Ti into crop soils through the application of various agrochemicals can increase the concentration of PTEs in soils over years of application [56,57]. The different sorghum crop sites (S1, S2, S3, and S4) showed Fe concentrations that exceeded the upper permissible limit, but lower Cu and Pb concentrations that were below the permissible limits established in the literature [54]. The high Fe concentrations may be from a natural source, such as the ashes and volcanic emissions expelled from the Popocatepetl volcano in recent years, or the incineration of municipal waste and in the mining area of the state of Morelos [54,58,59]. In addition, it has been reported that other chemical elements, such as Cd, Co, Cu, Mn, Ni and Zn, considered soil contaminants, are generated from mining and metallurgical activities near crops [52,53,60]. Coal, lead, and Zn mines are believed to be major sources of Fe, As, Cd, and Pb generation, which can contaminate soils adjacent to mines [60]. Both Mn and Fe are essential PTEs in agricultural fields, but at high concentrations, they can become contaminants in the surrounding soil and consequently cause health problems [58].

Regarding total PTEs concentration, the Tepoztlan crop site (S1) exhibited the highest concentration, followed by Yecapixtla (S2) and Tepalcingo (S3), and finally Xoxocotla (S4). These PTE concentrations were significantly different (*p* < 0.05): site S1 showed differences compared to sites S3 and S4, while site S2 showed differences compared to site S4. These results could be due to different sources of pollution, both anthropogenic and natural, namely, mining, thermoelectric plants, industrial zones, urban roads and highways, the use of agrochemicals, weathering and erosion phenomena, and volcanic ash. These sources of contamination are found close to the different sorghum cultivation sites in the state of Morelos (Figure 1) and could have varying effects on the different sites. For example, Tepoztlán (S1) is a town located near one of the busiest highways in Mexico, especially during weekends and holiday periods. The MEX-095D CDMX–Cuernavaca–Acapulco highway connects the capital of the country (Mexico City) with the state of Guerrero, passing right by Tepoztlán [61]. According to Gupta [62], in urban areas, vehicles emit PTEs through the combustion of diesel and gasoline, as well as through the wear of tires, brake pads, and engines, as well as corrosion of metal parts and paint. Furthermore, Tepoztlán is composed of various primary volcanic rocks, including lava, pyroclastic flows, and lahars [63], and weathering and erosion of these materials could occur and release potentially toxic compounds. In addition, although Tepoztlán and Yecapixtla are located a short distance from the high-risk zone of the Popocatepetl volcano, it is the latter locality that usually receives the largest amount of volcanic ash, which has been reported to be composed of PTEs such as As, Cd, Cr, Cu, Mn, Ni, Pb, and Zn [64]. Additionally, site S2 is located near an industrial area and a thermoelectric plant, which could contribute to the increased PTE levels at this site. Site S3, Tepalcingo, is located 10 km from the Siglo XXI highway, which connects the states of Puebla and Guerrero (MEX-095D highway) via the state of Morelos [65]. This highway is also one of the most important and heavily trafficked in the country, especially during holiday periods. Although less affected than sites S1 and S2, site S3 is also susceptible to volcanic ashfall. Site S4, on the other hand, is a connection point for the MEX-095D and Siglo XXI highways [66]. Furthermore, an industrial zone located 35 min from Xoxocotla and S4 it is also susceptible to volcanic ashfall, though to a lesser degree than the other sites.

Our results are consistent with those reported by Antoniadis et al. [40], who quantified traces of PTEs in soils collected in an industrial and volcanic area of Volos, Greece. They concluded that natural and anthropogenic sources can considerably affect soil contamination levels and consequently cause health risks.

If we only look at the top eight PTEs in terms of concentration, Fe showed the highest concentration in all the sites studied, followed by Mn and/or Ti. The rankings of the different PTEs are presented in Table 3.

The rankings in Table 3 are consistent with the results reported by Hossen et al. [43], who identified and quantified PTEs in 20 soil samples near a coal mining area in Barapukuria, Bangladesh. Their ranking was Fe (18,879 ± 8724 mg/kg) > Mn (226.12 ± 34.3 mg/kg) > Zn (101.96 ± 4.79 mg/kg) > Cr (82.37 ± 37.72 mg/kg) > Ni (56.54 ± 26.08 mg/kg) > Co (31.66 ± 9.86 mg/kg). The high concentrations and spatial differences between sampling sites were attributed to irrigation of the soils with water drained from the coal mine and their proximity to roads. However, the results obtained by these authors differ from those of the present study with respect to the high concentrations of the different PTEs. In this study, the different PTEs were present at concentrations within the limits established in the literature and do not represent a risk to the environment [67]. However, they can accumulate in soils and in the different tissues of sorghum plants and thus enter the food chain of humans and animals [2,13,68].

#### 3.2.2. Assessment of Soil Contamination Indices

**Figure 2:**
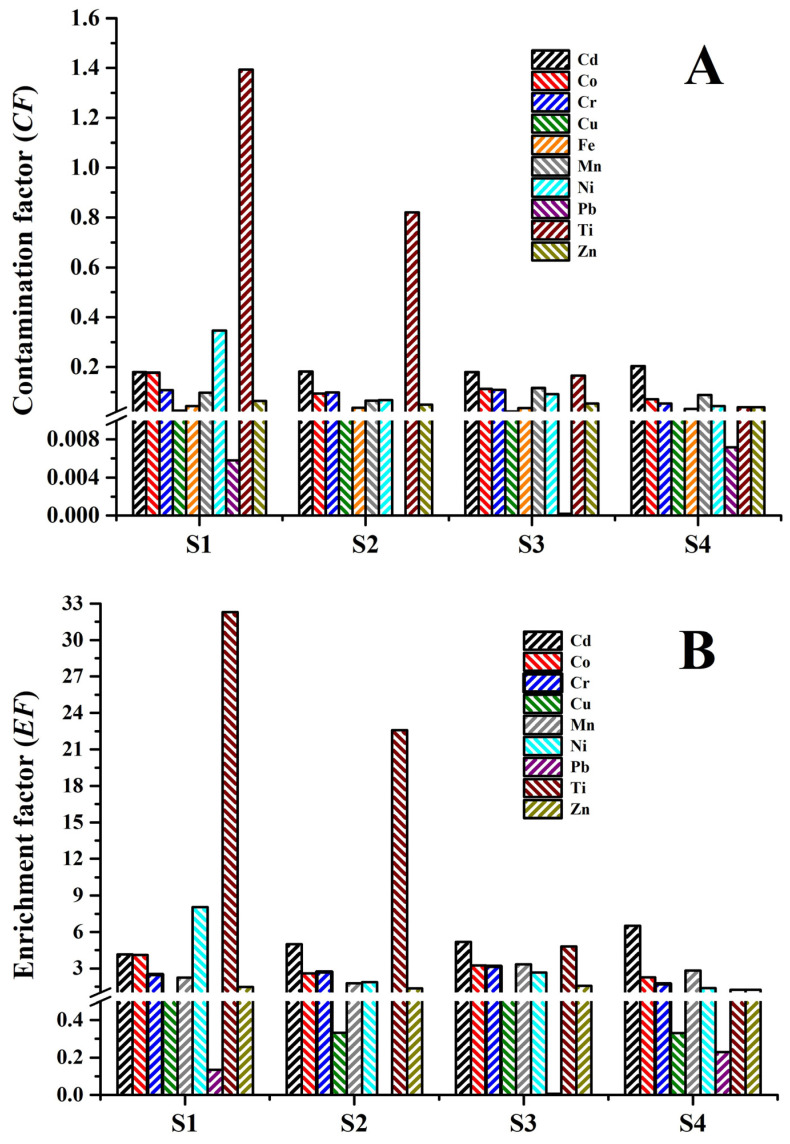
Soil contamination indices of the different sorghum cultivation sites: (**A**) contamination factor (*CF*) and (**B**) enrichment factor (*EF*).

The contamination factor (*CF*) and enrichment factor (*EF*) values obtained for the different sorghum cultivation sites studied are presented in Figure 2A,B. Only some PTEs were used to calculate the *CF* and *EF* values, namely Cd, Co, Cr, Cu, Mn, Ni, Pb, Ti, and Zn, because As presented *CF* and *EF* values close to 0, and Fe was used as a reference element [69]. The *CF* values were used to evaluate the level of PTE contamination in the soils. The PTEs can be ranked based on their average *CF* values as follows: Ti (0.61) > Cd (0.19) > Ni (0.14) > Co (0.12) > Cr (0.09) > Mn (0.09) > Zn (0.05) > Fe (0.04) > Cu (0.017) > Pb (0.003). According to the results, the soil of site S1 can be classified as moderately contaminated with Ti (CF value of 1.40), while sites S2, S3, and S4 exhibited values of 0.82, 0.17, and 0.04, respectively (Figure 2A). This result may be related to the incorporation of Ti into the crop soil due to the use of fertilizers for sorghum plant growth [56,57] or emissions from industries that produce paints and coatings with Ti and metallic materials containing titanium alloys [38]. Most soil samples showed values of CF ˂ 1 for all the PTEs evaluated, classifying them as pristine soils [39,40].

Figure 2B, which presents the *EF* values for the PTEs in the evaluated soils, shows that all the sorghum fields had *EF* values greater than 1. The PTEs can be ranked as follows: Ti (15.24) > Cd (5.22) > Ni (3.48) > Co (3.06) > Mn (2.56) > Cr (2.52) > Zn (1.51). These results indicate that these sorghum crop soils in Morelos are enriched with PTEs of anthropogenic origin. According to the classification based on *EF*, there is extreme (*EF* > 10) and severe enrichment (5 ≤ *EF* < 10) of Ti and Cd, respectively; there is moderate enrichment of Ni and Co (3 ≤ *EF* < 5) and there is lower enrichment of Mn, Cr, and Zn (1.5 ≤ *EF* < 3) [40,43,51]. These results indicate that the PTEs identified in the crop sites may be due to the use of fertilizers and pesticides on the crops, the industries that are near the sites, urban and industrial waste, as well as vehicle emissions [56,57,59].

Cu and Pb presented *EF* values ˂ 1 at all the crop sites (between 0.33 and 0.63, and 0 and 0.29, respectively), classifying the soils as standard soils (*EF* ˂ 1.5) for Cu and Pb. The presence of Cu and Pb at the different sites (Table 2) may be related to emissions from the active Popocatepetl volcano, industrial and mining activity, as well as highways in urban areas [3,6,59].

### 3.3. Accumulation of PTEs in Sorghum Plant Tissues

**Figure 3:**
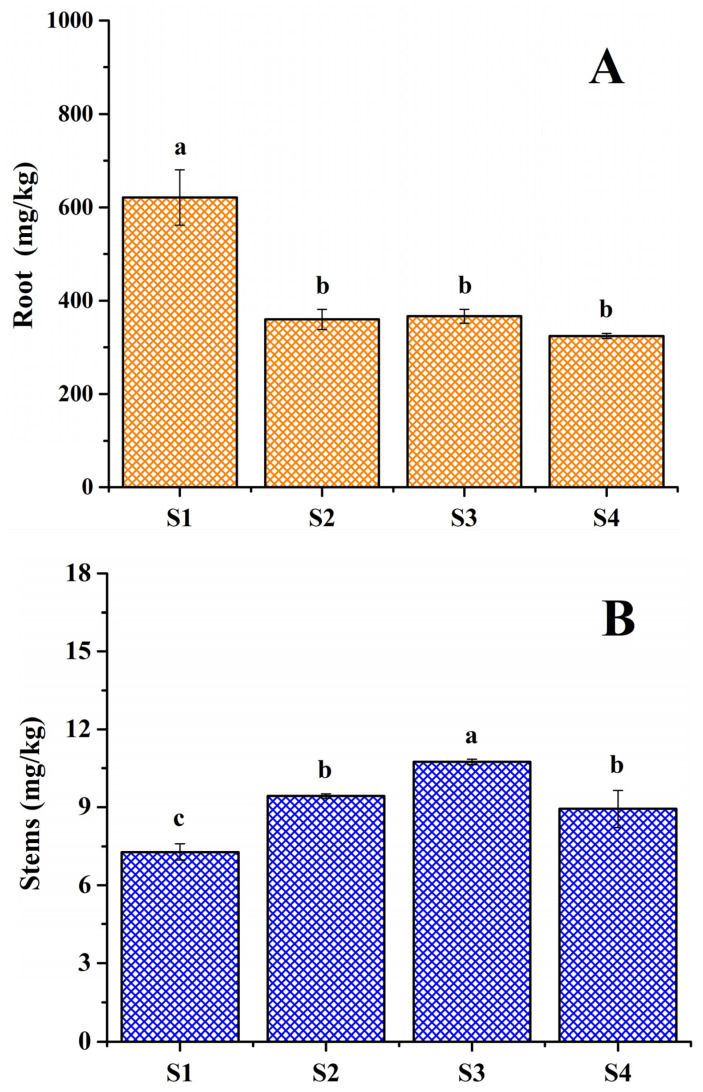

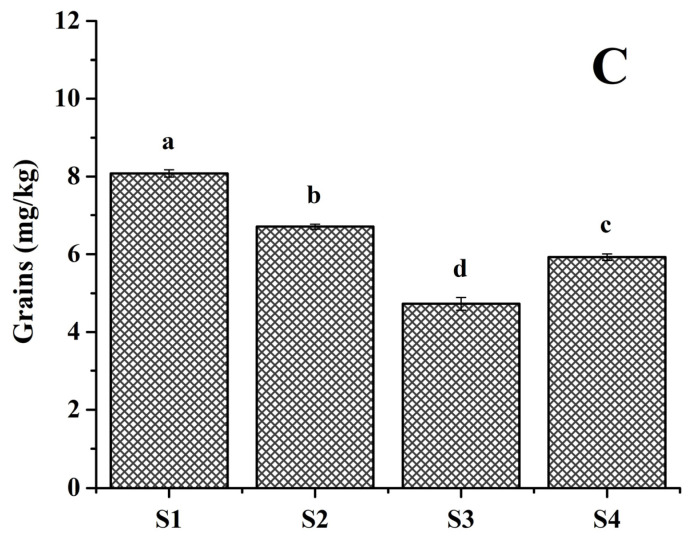
Concentration of PTEs in different parts of sorghum plants collected at the different study sites in the state of Morelos, Mexico: (**A**) roots, (**B**) stems, and (**C**) grains. Different letters above bars indicate significant statistical differences (*p* ˂ 0.05).

Among the sorghum plants collected at the four sites, roots presented the highest PTE concentrations, while stems and grains showed lower concentrations (Figure 3A–C). Generally, when soils contain high concentrations of PTEs, a higher concentration of PTEs is found in the root compared to other plant tissues [2,13,54,68,70]. In this study, the average total PTEs concentrations in the soils at sites S1, S2, S3, and S4 were 1441.7 ± 379, 1173.7 ± 320, 1098.2 ± 304, and 975.4 ± 276 mg/kg, respectively (Table 2), while the PTEs concentrations in the roots of sorghum plants collected at these sites were 620.9 ± 59, 360.1 ± 21, 366.8 ± 14, and 324.4 ± 5 mg/kg, respectively (Figure 3A). Site S1 showed the highest PTEs concentration compared to the other sites. The only significant differences in PTEs concentration (*p* < 0.05) were between site S1 and sites S2, S3, and S4. This result is consistent with those shown in Section 3.2.1 and agrees with the results reported in the literature [2,13,54,68,70].

In the root of the sorghum plants, the PTEs with the highest concentrations were Fe (295.8–557.4 mg/kg), Ti (1.8–34.1 mg/kg), Mn (9.6–18.3 mg/kg), Zn (1.9–5.3 mg/kg), and Cr (1.7–3.5 mg/kg). The PTEs with the lowest concentrations were Co (0.14–0.68 mg/kg), Cu (0.54–0.82 mg/kg), Ni (0.59–4.51 mg/kg), and Pb (0–0.023 mg/kg). According to the normal or phytotoxic PTE concentrations reported for trees and plants [71,72,73], the concentrations of Fe, Ti, and Ni in the roots of the sorghum plants exceeded the permissible limits for phytotoxicity. This result reveals that these plants showed more tolerance to Fe, Ti, and Ni compared to Mn, Zn, Cr, Co, Cu, and Pb. These results are consistent with the findings of Yuan et al. [13], who studied tissues the roots, stems, leaves, and grains of sweet sorghum plants, and they found the highest concentrations of Pb, Zn and Cd in the roots, while the stem presented the lowest concentrations. Likewise, they found that the concentrations of Pb and Zn were higher than those of Cd in most of the sweet sorghum plants studied; however, Pb and Zn concentrations were found to be at normal or permissible levels, while Cd showed a concentration higher than the reported phytotoxic level. Yuan et al. [13] concluded that sweet sorghum plants were more tolerant to Cd present in soils.

The total PTE concentration in the stems and grains of the sorghum plants is presented in Figure 3B,C, respectively. These concentrations in plants from the different crop sites showed significant differences (*p* ˂ 0.05) and were lower than those in the roots. For ease of interpretation, the percentages of the PTE concentrations transferred from the root to the stem of the sorghum plants were calculated: 1.2%, 2.6%, 2.9%, and 2.7% for sites S1, S2, S3, and S4, respectively. These results revealed that more than 97% of the PTEs remained in the roots of the sorghum plants, indicating that they cannot hyperaccumulate metals such as Co, Pb, Ni, and Ti [2]. The high percentage of PTEs in the roots of the sorghum plants can be attributed to their immobilization in the cell walls that make up the root tissues, or to the binding of PTEs by organic compounds present in the roots [2,70,73]. Our results are consistent with those reported by Pinto et al. [70], who studied the absorption and distribution of Cd in sorghum plants under controlled conditions. They found that Cd was retained mainly in the roots of sorghum plants, which was attributed to the immobilization of the metal by the cell walls in the roots.

The PTE levels in the grains of the sorghum plants from the different cultivation sites are presented in Figure 3C. These concentrations are lower than the concentrations in the stems of the plants but are on the same order of magnitude. The percentages of PTEs transferred from the root to the grains were calculated to be 1.3%, 1.86%, 1.28%, and 1.82% for sites S1, S2, S3, and S4, respectively. The PTEs with the highest concentrations in the stems were Fe (4.565 mg/kg), Zn (2.461 mg/kg), Mn (1.791 mg/kg), and Ni (0.287 mg/kg); the average concentrations of these PTEs in grains were 3.215, 1.441, 1.173, and 0.134 mg/kg, respectively. The percentages of Fe, Zn, Ni, and Mn transferred from the stems to the grains of the sorghum plants were 70.42%, 58.57%, 46.69%, and 65.49%, respectively. These results indicate that most of these PTEs were transferred to the grains (aerial parts) of the plants. Zhuang et al. [46] studied removal of metals by sorghum plants, namely, the Pb, Cd, Zn, and Cu present in contaminated soils; they discovered that these heavy metals were transferred from the root to the stems and aerial parts of the plants, with higher absorption of Pb in the leaves and a higher concentration of Cd, Zn, and Cu in the stems. These findings promoted the use of sorghum plants as an alternative strategy in the development of phytoextraction technology.

In other plants, it has been reported that during the process of uptake and transfer of PTEs (essential and non-essential) by the different plant tissues involves several stages; (1) uptake of metals through the roots and transfer to xylem tissues; (2) transfer of PTEs from the root to the shoots by xylem flow, which can be redirected through intervascular transfer at the nodes; and (3) mobilization of metals from leaf ducts to grains through the phloem [2,74]. These stages are a function of the concentration gradient of PTEs, as well as the diffusion and selective uptake capacity of each metal in the different plant tissues. The level of uptake and transfer of PTEs can differ between different plant species and due to interactions between PTEs and compounds (molecules, fibrils, etc.) present in plant tissues [51,75].

### 3.4. Contamination Assessment Indices in Sorghum Plants

#### 3.4.1. Bioaccumulation Factor

**Figure 4:**
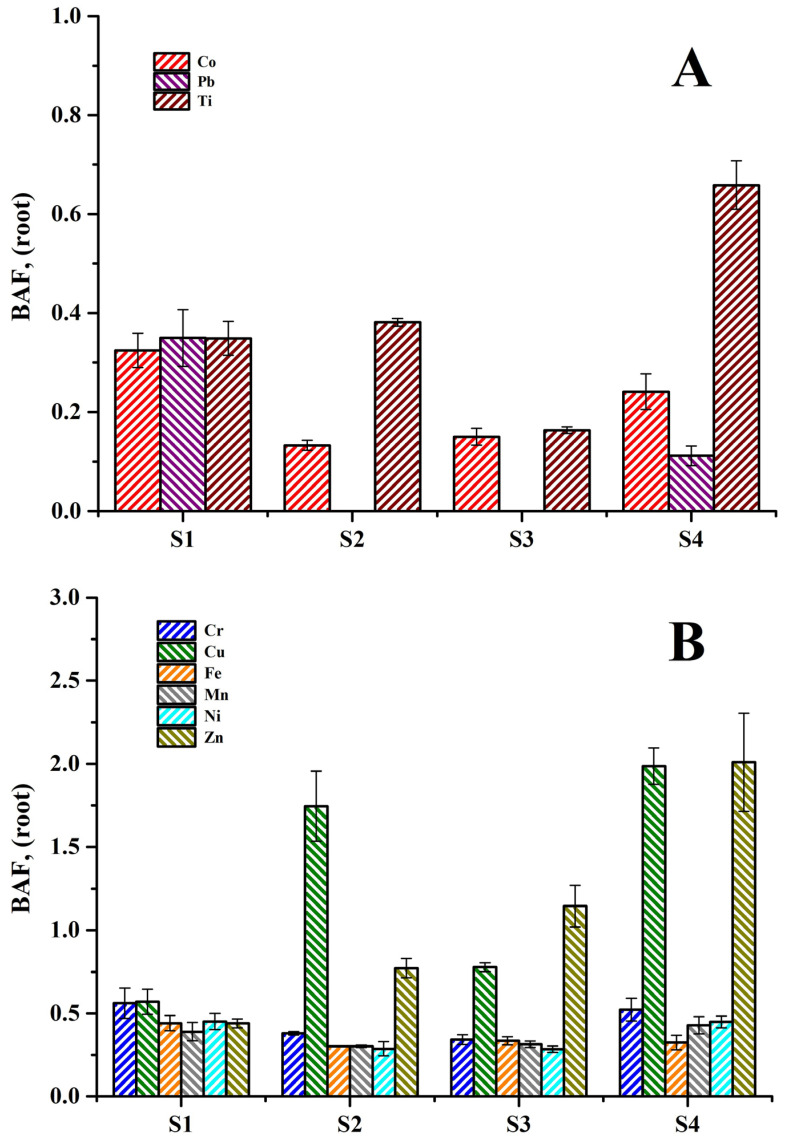

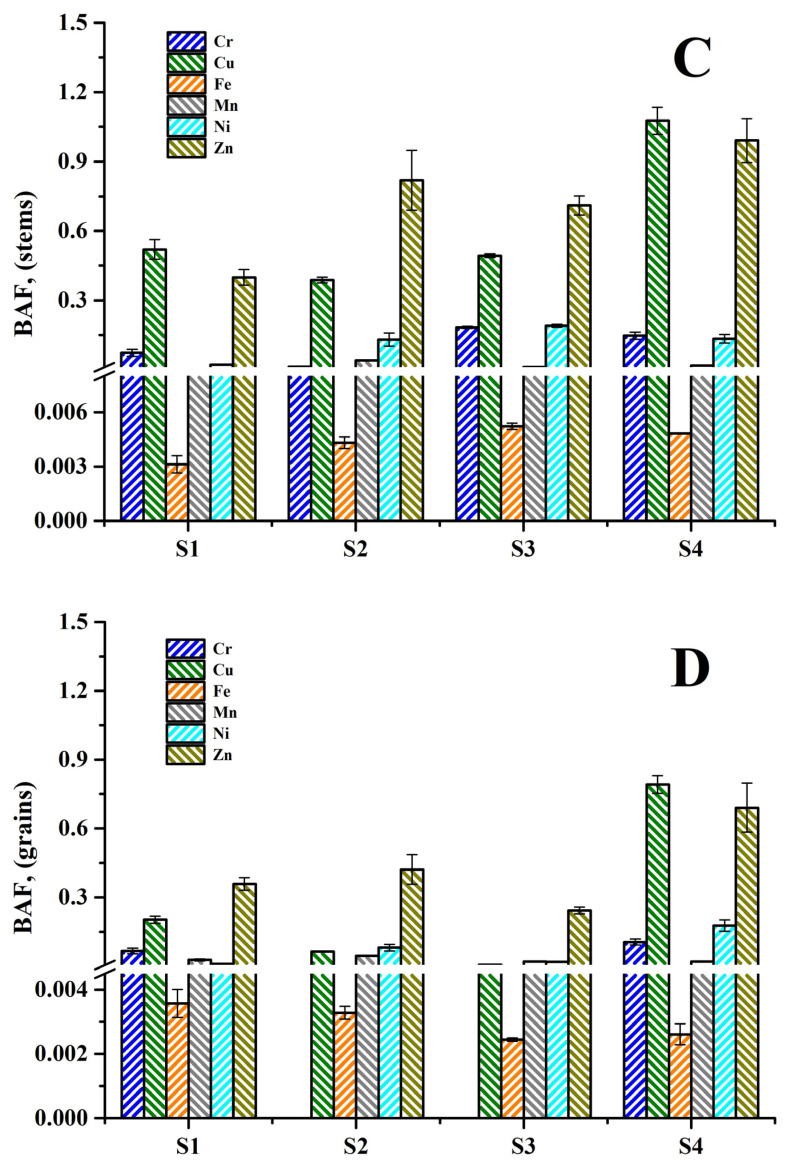
Bioaccumulation factor (*BAF*) values of PTEs for transfer from the soil to different plant tissues: (**A**) soil to roots for Co, Pb, and Ti; (**B**) soil to roots; (**C**) soil to stem; (**D**) soil to grains.

The transfer of PTEs from soil to plant tissues is a key process in the exposure of PTEs to humans and animals through the food chain [46]. Thus, the bioaccumulation factor (*BAF*) values for the transfer of PTEs from crop soils to the roots, stem, and grains of sorghum plants were calculated and are presented in Figure 4A–D. Figure 4A shows the *BAF* values for the transfer of Co, Pb, and Ti from the soil to roots, which ranged from 0.13 to 0.33, 0 to 0.35, and 0.16 to 0.66, respectively. Therefore, these PTEs can be ranked as Ti > Co > Pb. It is important to mention that Co, Pb, and Ti were only identified and quantified in the crop soils and roots of the sorghum plants because these PTEs were not transferred to the stems or aerial parts. These PTEs exhibited *BAF* values ˂ 1, indicating that the plants only absorbed these metals but did not accumulate them [13,44]. The *BAF* values for the transfer of Cr, Cu, Fe, Mn, Ni, and Zn from the soil to the roots are shown in Figure 4B. Zn (0.44–2.01) and Cu (0.57–1.98) showed high *BAF* values, while relatively low values were obtained for Cr (0.34–0.56), Ni (0.29–0.45), Fe (0.30–0.45), and Mn (0.31–0.43). Thus, these PTEs can be ranked as Zn > Cu > Cr > Ni > Fe > Mn. A *BAF* > 1 indicates that the plant accumulates that metal in its tissues [44]. Sites S1, S2, and S3 showed Cu and Zn *BAF* values greater than one, while the other PTEs showed *BAF* values less than one (Figure 4B). This result indicates that sorghum plants have a higher root accumulation capacity for Cu and Zn compared to Cr, Ni, Fe, and Mn. Cu and Zn are essential micronutrients for plant growth and development, actively participating in photosynthesis, respiration, and other metabolic processes. Therefore, the *BAF* values > 1 for Cu and Zn may be due to the different solubilities of these PTEs in soils, which regulate their availability for sorghum plants [41,76].

The *BAF* values for the transfer of PTEs from soil to stems and from soil to grains of the sorghum plants are presented in Figure 4C,D, respectively. Figure 4C shows that the *BAF* values for Cu (0.39–1.08) and Zn (0.40–1.00) were higher, while relatively low values were found for Ni (0.02–0.19), Cr (0.015–0.19), Mn (0.009–0.040), and Fe (0.003–0.005). Thus, these PTEs can be ranked as follows: Cu > Zn > Ni > Cr > Mn > Fe. Figure 4C also shows that Cu and Zn showed *BAF* values greater than one at site S4, while the rest of the PTEs at site S4 as well as all the heavy metals at sites S1, S2, and S3 presented values less than one. According to Satpathy et al. [44], this finding establishes that sorghum plants accumulate Cu and Zn in their stem tissues. This observation may be due to the lower concentration of PTEs in the roots at site S4 compared to the concentrations of PTEs in the roots of plants collected at sites S1, S2, and S3 (Figure 3A).

Finally, Figure 4D shows the values of *BAF* for PTEs transferred from the soil to the grains of sorghum plants at different cultivation sites. None of the PTEs identified and quantified in the grains exhibited *BAF* values > 1 at any of the sites. This indicates that the grains only absorbed these PTEs and did not accumulate them.

Regarding toxicity, according to FAO/WHO [48], the maximum permitted level in food is 0.05–0.5 mg/kg for Cu, less than 0.8 mg/kg for Fe, and 0.3–1 mg/kg for Zn. The PTE concentrations found in the grains of sorghum plants in this study were 0.03–0.32 mg/kg for Cu, 2.38–4.49 mg/kg for Fe, and 0.91–1.83 mg/kg for Zn, which are equal to or higher than the permissible limits in international food standards [48].

#### 3.4.2. Translocation Factor

The translocation factor (*TF*) values of the PTEs for translocation from soil to sorghum roots (*TF*_S-R_), roots to stem (*TF*_R-T_), and roots to grains (*TF*_R-G_) are presented in Table 4. The average *TF*_S-R_ values can be ranked as follows: Cu (1.27 ± 0.7) > Zn (1.09 ± 0.7) > Cr (0.45 ± 0.1) > Ti (0.39 ± 0.2) > Ni (0.37 ± 0.1) > Mn (0.36 ± 0.06) > Fe (0.35 ± 0.06) > Pb (0.23 ± 0.2) > Co (0.21 ± 0.09). The average *TF*_R-T_ values can be ranked as follows: Zn (0.77 ± 0.26) > Cu (0.58 ± 0.29) > Ni (0.37 ± 0.26) > Cr (0.25 ± 0.22) > Mn (0.06 ± 0.05) > Fe (0.013 ± 0.004). Co, Pb, and Ti showed *TF*_R-T_ values close to zero. The *TF*_R-G_ values can be ranked as follows: Zn (0.48 ± 0.26) > Ni (0.19 ± 0.1) > Mn (0.083 ± 0.04). Almost all the heavy metals showed significant differences in *TF*_S-R_ values (*p* < 0.05) between the four sites, except for Mn; the Pb results could not be subjected to a complete analysis of variance since it was only detected in the soil and roots of the plants at S1 and S4.

The translocation factor values of the PTEs for translocation from the soil to plant roots can be used to determine the exposure of humans to these metals through the food chain and reveal the bioavailability of PTEs in the soils. Higher *TF* values indicate higher mobility and/or availability of PTEs [44,77]. The PTEs can be ranked based on average TF_R-G_ values as follows: Zn (0.48 ± 0.26) > Ni (0.19 ± 0.1) > Mn (0.083 ± 0.04). All these values are less than 1, indicating a low level of translocation of these PTEs, which implies that they were retained in the roots of the sorghum plants. However, the average values for Fe, Zn, Ni and Mn in the grains were, respectively, 4.5, 1.59, 0.11, and 1.29 mg/kg at S1; 3.5, 1.43, 0.16, and 1.44 mg/kg at S2; 2.5, 0.92, 0.05, and 1.12 mg/kg at S3; and 2.38, 1.83, 0.22, and 0.85 at S4. The higher PTEs concentrations in the grains of the sorghum plants compared to their calculated translocation values (TF_R-G_) could indicate that the sorghum plants were contaminated with PTEs from the air, which could be from volcanic emissions, the use of agrochemicals on crops, industries near the cultivation area, urban and industrial waste, as well as vehicle emissions [56,57,59].

## 4. Conclusions

In this study, potentially toxic elements (PTEs) present in sorghum soils were quantified at four sites (S1, S2, S3, and S4) in central Mexico, as well as the transfer of PTEs from the soil to the tissues (root, stem, and grains) of the sorghum plants. The following conclusions were drawn. The PTEs with the highest concentrations in the crop soils were Fe (1272.0–921.0 mg/kg) > Mn (56.2–31.6 mg/kg) > Ti (98.1–3.0 mg/kg). The concentrations of Fe present in the soils exceeded the permitted limit, while Mn and Ti showed concentrations below the permitted limits. The high concentrations of Fe, Mn, and Ti are related to volcanic emissions and the proximity to the industrial and mining area of central Mexico. In addition, these PTEs can accumulate in the soil as well as in the different tissues of sorghum plants. Based on enrichment factor (*EF*) values, the PTEs can be ranked as Ti > Cd > Ni > Co > Mn > Cr > Zn, and the values indicate that the sorghum soils were contaminated with PTEs of anthropogenic origin. Cu and Zn showed bioaccumulation factor (*BAF*) values > 1 in the roots and stems of sorghum plants collected at sites S1 and S2, indicating that the plants accumulated Cu and Zn in their stems. The PTEs with the highest concentrations in the stems of the sorghum plants were Fe (4.56 mg/kg), Zn (2.46 mg/kg) and Mn (1.79 mg/kg); in the grains, they were Fe (3.22 mg/kg), Zn (1.44 mg/kg), Mn (1.17 mg/kg) and Cu (0.03 to 0.32 mg/kg). According to the FAO, the maximum permitted levels of toxic contaminants in food are 0.05 to 0.5 mg/kg for Cu, ˂0.8 mg/kg for Fe, and 0.3 to 1 mg/kg for Zn. The concentrations of these PTEs in the grains of the sorghum plants were 0.137 ± 0.04, 3.215 ± 0.99, and 1.441 ± 0.38 mg/kg, respectively, which are higher than the permitted limits in international food standards. Finally, Zn, Ni, and Mn showed translocation (TF) values of less than 1, which implies that these PTEs were retained in the roots of the sorghum plants. However, the average Fe, Zn, Ni, and Mn concentrations in the grains were higher than the TF_R-G_ values of these PTEs, indicating that the sorghum plants may have been contaminated with PTEs from the air, which could be attributed to volcanic emissions, the use of agrochemicals on the crops, industries near the cultivation area, urban and industrial waste, and vehicle emissions. To our knowledge, this study constitutes the first report on the identification, quantification, and assessment of PTE translocation in sorghum plants grown under open-field conditions near urban-volcanic contamination sources.

## Figures and Tables

**Table 1:** Chemical characteristics of different sorghum growing soils in the state of Morelos, Mexico.

Parameter	S1	S2	S3	S4
pH	4.9	5.5	6.6	5.8
OM (%)	1.75	2.34	2.34	4.09
CEC (Cmol/kg)	10.4	14.0	11.0	17.2

**Table 2:** Concentration of PTEs identified in different sorghum crop soils.

PTE	S1	S2	S3	S4	Safe Values *	Permissible Limit **
As	---	0.1 ± 0.02	0.12 ± 0.01	0.05 ± 0.04	20	6.83
Cd	0.1 ± 0.04	0.1 ± 0.007	0.07 ± 0.004	0.1 ± 0.006	1	0.41
Co	2.0 ± 0.3	1.07 ± 0.06	1.3 ± 0.03	1.0 ± 0.1	20	11.3
Cr	6.4 ± 1.3	6.0 ± 0.39	7.0 ± 0.16	3.21 ± 0.43	100	59.5
Cu	0.95 ± 0.07	0.5 ± 0.02	0.1 ± 0.01	0.4 ± 0.02	100	38.9
Fe	1272.2 ± 164.0	1071.4 ± 73.0	1015 ± 40.0	921 ± 115.0	---	425
Mn	47.4 ± 7.7	31.6 ± 1.6	56.2 ± 2.2	43.0 ± 5.0	---	488
Ni	10.04 ± 1.5	1.95 ± 0.36	2.7 ± 0.1	1.2 ± 0.1	50	29
Pb	0.2 ± 0.01	---	---	0.2 ± 0.02	50	27
Ti	98.1 ± 11.9	58.0 ± 2.2	12.0 ± 0.3	3.0 ± 0.2	---	7038
Zn	4.4 ± 0.03	3.1 ± 0.5	4.0 ± 0.2	3.0 ± 0.4	200	70
Total	1441.7 ± 379 ^a^	1173.7 ± 320 ^ab^	1098.2 ± 304 ^bc^	975.4 ± 276 ^c^		

Data are shown as means ± standard deviations (mg/kg). --- The elements were not detected by the measuring equipment. Different letters in the last row indicate significant statistical differences (*p* ˂ 0.05). * From [48]; ** from Kabata-Pendias and Pendias [38].

**Table 3:** Ranking of PTEs concentrations in sorghum crop soils.

Site	PTEs
S1	Fe > Ti > Mn > Ni > Cr > Zn > Co > Cu
S2	Fe > Ti > Mn > Cr > Zn > Ni > Co > Cu
S3	Fe > Mn > Ti > Cr > Zn > Ni > Co > Cu
S4	Fe > Mn > Cr > Ti > Zn > Ni > Co > Cu

**Table 4:** Translocation factor values for PTEs identified in crop soils compared to different sorghum plant tissues.

		Co	Cr	Cu	Fe	Mn	Ni	Pb	Ti	Zn
S1	*TF*_S-R_	0.32 ± 0.1 ^a^	0.56 ± 0.1 ^a^	0.57 ± 0.1 ^b^	0.44 ± 0.1 ^a^	0.39 ± 0.1 ^a^	0.45 ± 0.1 ^a^	0.35 ± 0.1	0.35 ± 0.0 ^b^	0.44 ± 0.01 ^c^
	*TF*_R-T_	---	0.13 ± 0.01	0.92 ± 0.05	0.01 ± 0.01	0.02 ± 0.01	0.05 ± 0.01	---	---	0.91 ± 0.03
	*TF*_R-G_	---	0.12 ± 0.04	0.36 ± 0.03	0.01 ± 0.00	0.07 ± 0.01	0.02 ± 0.01	---	---	0.82 ± 0.05
S2	*TF*_S-R_	0.13 ± 0.0 ^c^	0.38 ± 0.0 ^b^	1.75 ± 0.2 ^a^	0.3 ± 0.0 ^b^	0.30 ± 0.01 ^a^	0.29 ± 0.1 ^b^	---	0.38 ± 0.0 ^b^	0.77 ± 0.1 ^bc^
	*TF*_R-T_	---	0.04 ± 0.01	0.22 ± 0.02	0.01 ± 0.01	0.13 ± 0.01	0.45 ± 0.04	---	---	1.06 ± 0.1
	*TF*_R-G_	---	---	0.04 ± 0.01	0.01 ± 0.00	0.15 ± 0.01	0.29 ± 0.03	---	---	0.54 ± 0.05
S3	*TF*_S-R_	0.15 ± 0.02 ^c^	0.34 ± 0.03 ^b^	0.78 ± 0.03 ^b^	0.36 ± 0.03 ^b^	0.31 ± 0.02 ^a^	0.29 ± 0.02 ^b^	---	0.16 ± 0.01 ^c^	1.15 ± 0.13 ^b^
	*TF*_R-T_	---	0.54 ± 0.03	0.66 ± 0.02	0.02 ± 0.01	0.04 ± 0.01	0.67 ± 0.02	---	---	0.62 ± 0.03
	*TF*_R-G_	---	---	0.01 ± 0.00	0.01 ± 0.00	0.06 ± 0.00	0.07 ± 0.01	---	---	0.21 ± 0.02
S4	*TF*_S-R_	0.24 ± 0.04 ^b^	0.52 ± 0.01 ^ab^	1.99 ± 0.11 ^a^	0.33 ± 0.04 ^b^	0.43 ± 0.05 ^a^	0.45 ± 0.04 ^a^	0.11 ± 0.1	0.66 ± 0.03 ^a^	2.01 ± 0.3 ^a^
	*TF*_R-T_	---	0.28 ± 0.01	0.54 ± 0.03	0.02 ± 0.002	0.043 ± 0.001	0.30 ± 0.02	---	---	0.50 ± 0.03
	*TF*_R-G_	---	0.20 ± 0.01	0.40 ± 0.02	0.14 ± 0.06	0.05 ± 0.00	0.40 ± 0.02	---	---	0.34 ± 0.02

Different letters in the PTE columns indicate statistically significant differences (*p* ˂ 0.05) in *TF*_S-R_. --- values were not calculated.

## Data Availability

The original contributions presented in this study are included in the article/Supplementary Materials. Further inquiries can be directed to the corresponding author.

## Supplementary Materials

The following supporting information can be downloaded at: https://www.mdpi.com/article/10.3390/toxics14040290/s1, Table S1. ICP OES 8300 DV operating conditions and acquisition parameters. Table S2. LOD/LOQ for examined PTEs.

## Abbreviations

The following abbreviations are used in this manuscript:

PTEs	Potentially toxic elements
Ca	Calcium
Cu	Copper
Fe	Iron
Mg	Magnesium
Mn	Manganese
Zn	Zinc
As	Arsenic
Cd	Cadmium
Pb	Lead
Cr	Chromium
Co	Cobalt
Ni	Nickel
Ti	Titanium
OM	Organic matter
CEC	Cation exchange capacity
ICP-OES	Inductively coupled plasma optical emission spectroscopy
CF	Contamination factor
EF	Enrichment factor
BAF	Bioaccumulation factor
TF	Translocation factor
