# Supplementary material for: Identification and Translocation of Potentially Toxic Elements in Sorghum Plants Grown in Central Mexico
Source: Toxics. 2026 Mar 28;14(4):290. doi: 10.3390/toxics14040290 (PMC13119858; doi:10.3390/toxics14040290)
Supplement: Supplementary file 1 [file toxics-14-00290-s001.zip › toxics-4188561-supplementary.pdf]

Supplementary material

The sampling, preparation and analysis of heavy metals in the soil samples were carried out under a system of assurance (QA) and quality control (QC) in accordance with the technical guidelines established in recognized international methodologies. In addition, from sampling to instrumental analysis, a safeguard of the samples was maintained, ensuring their traceability and integrity. Sample preparation was carried out by acid plate digestion (Cimarec, Thermo Scientific, Massachusetts, U.S.A). Metal quantification was performed using ICP-OES (8300 DV, Perkin Elmer, Massachusetts, U.S.A), with daily calibrations and verification of the instrument after analysis of every 10 samples.

On the other hand, quality control included the evaluation of targets, certified reference materials, triplicates, and fortified samples. The targets of the reagents and digestion presented concentrations below the detection limit of the method, ruling out external contamination. The analytical accuracy of the soil samples was verified by analysis of certified reference material (NIST SRM 2711a, Montana II Soil). Also, recoveries for the metals tested were within the 80 - 120% acceptance range. Accuracy was evaluated by triplicate digestion, obtaining relative standard deviation (RSD) values < 15%.

The recoveries of the fortified samples ranged from 88 to 105%, confirming the efficiency of the digestion process and the absence of significant interference from the soil matrix. The calibration curves presented coefficients of determination (R^2) > 0.995.

On the other hand, the limits of detection (LOD) and quantification (LOQ) were adequate to assess compliance with the maximum permissible limits established in the applicable NOMs. All reported analytical results met the acceptance criteria defined in the Q_A/Q_C system.

Table S1. ICP OES 8300 DV operating conditions and acquisition parameters.

Operating conditions	Values
**Instrument**	ICP OES 8300 DV
**Cone**	Cones are not used
**Auxiliary gas flow**	1 L/min
**Nebulizer gas flow**	1 L/min
**Peristaltic pump flow**	1 mL/min
**He flow**	12 mL/min
**Rf power**	1300 watts

Table S2. LOD/LOQ for examined PTEs.

Element	Wavelength	LOD mg/l	LOQ mg/l
**Cr**	283.563	0.002	0.02
**Ni**	231.604	0.005	0.05
**Fe**	238.204	0.002	0.02
**Mn**	257.610	0.0004	0.004
**Zn**	206.200	0.001	0.01
**Cu**	327.393	0.004	0.04
**Cd**	228.802	0.001	0.01
**Pb**	220.353	0.01	0.1
**As**	188.979	0.05	0.5
**Co**	228.616	0.001	0.01
